# Impact of Fermentation on the Phenolic Compounds and Antioxidant Activity of Whole Cereal Grains: A Mini Review

**DOI:** 10.3390/molecules25040927

**Published:** 2020-02-19

**Authors:** Oluwafemi Ayodeji Adebo, Ilce Gabriela Medina-Meza

**Affiliations:** 1 Department of Biotechnology and Food Technology, Faculty of Science, University of Johannesburg, Doornfontein Campus, P.O. Box 17011, Gauteng, South Africa; 2 Department of Biosystems and Agricultural Engineering, Michigan State University, 524 South Shaw Lane, East Lansing, MI 48824-1323, USA; ilce@msu.edu

**Keywords:** fermentation, fermented foods, whole grains, health benefits, phenolic compounds, antioxidant activity

## Abstract

Urbanization, emergence, and prominence of diseases and ailments have led to conscious and deliberate consumption of health beneficial foods. Whole grain (WG) cereals are one type of food with an array of nutritionally important and healthy constituents, including carotenoids, inulin, β-glucan, lignans, vitamin E-related compounds, tocols, phytosterols, and phenolic compounds, which are beneficial for human consumption. They not only provide nutrition, but also confer health promoting effects in food, such as anti-carcinogenic, anti-microbial, and antioxidant properties. Fermentation is a viable processing technique to transform whole grains in edible foods since it is an affordable, less complicated technique, which not only transforms whole grains but also increases nutrient bioavailability and positively alters the levels of health-promoting components (particularly antioxidants) in derived whole grain products. This review addresses the impact of fermentation on phenolic compounds and antioxidant activities with most available studies indicating an increase in these health beneficial constituents. Such increases are mostly due to breakdown of the cereal cell wall and subsequent activities of enzymes that lead to the liberation of bound phenolic compounds, which increase antioxidant activities. In addition to the improvement of these valuable constituents, increasing the consumption of fermented whole grain cereals would be vital for the world’s ever-growing population. Concerted efforts and adequate strategic synergy between concerned stakeholders (researchers, food industry, and government/policy makers) are still required in this regard to encourage consumption and dispel negative presumptions about whole grain foods.

## 1. Introduction

Foods in the past were known to conventionally provide nutrients necessary for basic physiological functions. This assumption has changed with available knowledge at the disposal of consumers, changes in food regulations, and an ever-growing health-conscious population, which are factors resulting in an increasing desire for foods with additional physiological benefits. The 2500-year-old concept of “Let food be thy medicine and medicine be thy food” by Hippocrates is now being embraced better than ever as consumers are gradually becoming aware of the importance of diet in health promotion and disease prevention. Such a concept of food as medicine could have led to the trend of what is now known as “functional foods,” which is a concept first created in Japan in the 1980s [1].

Supporting this perspective of food as medicine are several studies on whole grains (WGs) and WG-diets having positive effects on disease markers such as blood pressure, diabetes, and obesity [2,3,4,5,6,7,8,9,10,11]. WGs are essentially made up of the germ, bran, and endosperm and contains all the important parts of the entire grain seed in their original proportions. A more detailed and approved definition by the American Association of Cereal Chemists (AACC) says “WGs shall consist of the intact, ground, cracked, or flaked caryopsis, whose principal anatomical components—the starchy endosperm, germ, and bran—are present in the same relative proportions as they exist in the intact caryopsis” [12]. On the contrary, refined grains (RGs) are products obtained after the refining process involving the removal of the most potent protective components of the grains found in the bran and germ. This consequently leaves only the starchy-rich endosperm. The retained protective components in WGs make them better constituents of beneficial components as compared to their refined counterparts.

Health beneficial constituents of WGs include phytochemicals, bioactive carbohydrate fractions, peptides, and other phytonutrients [11,13,14,15,16]. WGs contain high amounts of phytochemicals, which are plant secondary metabolites that have shown biological activity and have been broadly investigated as health beneficial groups of compounds in food [17,18,19]. Particularly important are phenolic constituents, which are major forms of these phytochemicals and vital with reference to their unique contribution to the health benefits of WGs. The major sources of these phytochemicals are phenolic compounds (PCs) due to the high concentrations of bioactive constituents in the bran and germ layer [17,20,21] and the fact that they are largely one of the most important dietary sources of energy intake worldwide.

## 2. Phenolic Compounds in WG Foods

**Figure 1:**
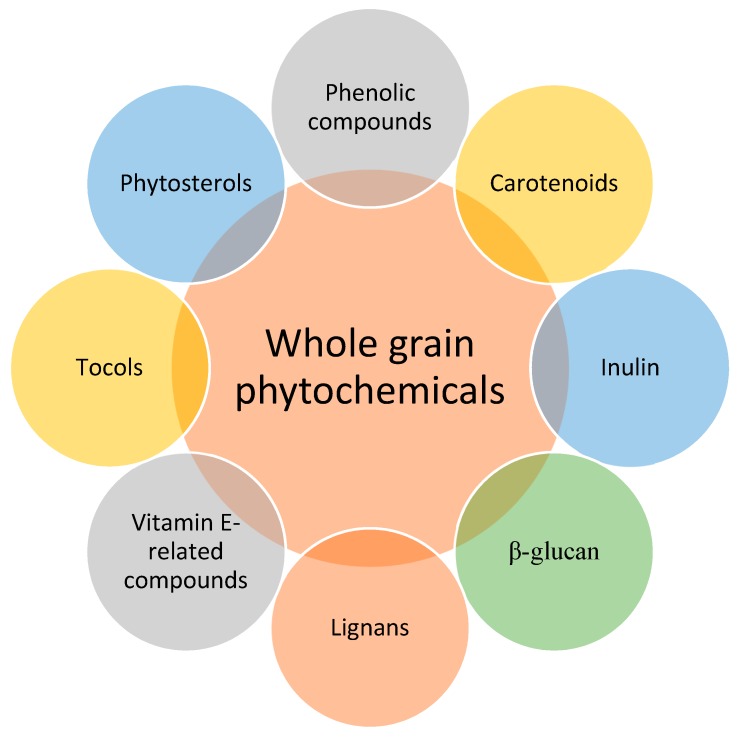
Whole grain phytochemicals.

The overall benefit derived from three major components of WG (germ, bran, and endosperm) altogether is higher than any of the individual fractions [22,23]. A combination of these components makes WG contain physiologically important components including vitamins, fatty acids, phytosterols, PCs, fatty acids, dietary fiber, carotenoids, lignans, and sphingolipids (Figure 1), which can promote health either singly or in synergy with each other [18,24]. A series of meta analyses and multiple scientific studies have equally reported an association between increasing intake of WG-foods and reduced risk of non-communicable diseases such as cardiovascular diseases, coronary heart diseases, stroke [24,25,26], metabolic syndrome [27], and cancers [28,29] as well as a positive effect on gut microbiota [30]. Phenolic compounds are subsequently discussed in this review as it is of vital importance in WG-cereals [16] and the fact that they are the most studied phytochemicals [31]. Usually, WGs may be consumed as food after it has been incorporated as an ingredient into other food products or as food itself after processing. One type of such a food processing technique adopted for the transformation of WGs into diets is fermentation, which is a process that yields products that are not only shelf stable, but also better in sensorial qualities and health beneficial constituents [32,33,34,35,36]. The cereal bran is a major source of these PCs and this paper seeks to review available scientific literature on fermented WG-products to understand the influence and role of fermentation on PCs and antioxidant activity (AA) thereof.

**Figure 2:**
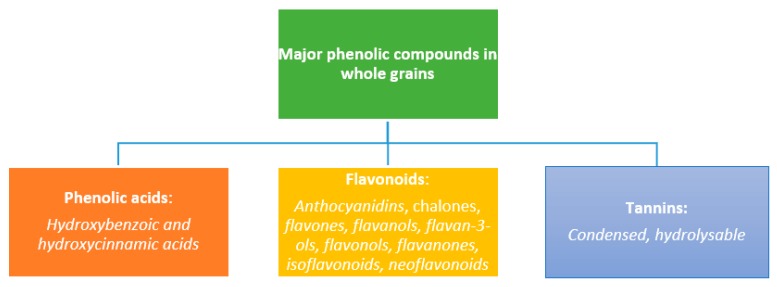
Classification of major phenolic compounds in whole grains.

Phenolic compounds (also called phenolics) are derived from several biosynthetic precursors including pyruvate, acetate, some amino acids (phenylalanine and tyrosine), malonyl CoA, acetyl CoA through the action of pentose phosphate, shikimate, and phenylpropanoid metabolism pathways [37,38,39]. The term ‘phenolic acids’ refers to phenolic compounds having one carboxylic acid group and are mainly divided into two subgroups, i.e., hydroxybenzoic acids (such as gallic, p-hydroxybenzoic, protocatechuic, syringic, and vanillic acids) and hydroxycinnamic acids (caffeic, ferulic, p-coumaric, and sinapic acids) (Figure 2). Flavonoids are an equally well-known class of frequently occurring phenolics in WGs. Major phenolics found in WGs are phenolic acids (PAs), flavonoids, and tannins. These plant-derived constituents are bioactive and involved in potentiating the redox defense of the body, prevention, and counteracting oxidative stress and reducing free radical-related cellular damage.

As stated by Singh et al. [40], flavonoids are the largest group of phenolics and account for the half of known PCs in plants. These compounds are equally low molecular weight compounds consisting of two aromatic rings (A and B) joined by a three-carbon bridge (C_6_–C_3_–C_6_ structure) [40]. Tannins, on the other hand, are high molecular weight polymeric phenolic compounds known to contribute to the pericarp (seed coat) color of cereals. These polyphenolic compounds have molecular weights of between 500–3000 g/mol, containing sufficient hydroxyls and other groups including carboxyl [41,42,43]. Tannins can be broadly classified into two, which include hydrolysable tannins [esters of ellagic acid (ellagitannins) or gallic acid (gallotannins)] and condensed tannins [(called polymeric proanthocyanidins) and known to be composed of flavonoid units) [41,44]. A plethora of excellent reviews and scientific literature are available in the literature on detailed classifications, forms, occurrences, and formation/generation of these compounds [15,16,40,41,45,46,47,48,49,50].

## 3. Fermentation of WG Foods

Food processing is essential for the transformation of food crops into edible forms. Fermentation is an old food processing technique that has been adopted for centuries around the world, especially in developing nations. It involves an intentional conversion/modification of a substrate through activities of microorganisms to get a desired product. This is usually completed through microbial actions, which positively alter the appearance, flavor, functionalities, nutritional composition, color, and texture. The fermentation process itself yields beneficial effects through direct microbial action and production of metabolites and other complex compounds [51,52,53]. Conventional techniques of fermentation include (i) natural (also called spontaneous) occurrences through the actions of endogenous microorganisms, (ii) back slopping involves utilizing plenty of successful previous fermentation batches) and (iii) controlled fermentation, which entails the inoculation of starter cultures/specific strains. Subsequent fermented products are not only shelf stable through the preservative effect of this process, but fermentation also improves bioavailability and palatability, confers desirable organoleptic characteristics that impact aroma, texture, and flavor and improves the health beneficial components in food [32,33,34,35,36]. Irrespective of the food substrate (cereal, legume, vegetable, fruit, RG, or WG), fermentation results in the modification of inherent constituents, secondary metabolites, detoxification of toxic components/residues, and improvement in the functionality of the food product [35,36,53,54,55].

The incorporation of WG into diet which, is influenced by cultural beliefs, disadvantages of longer cooking time, the presence of phytates, tannins, and a limited variety of products made from them [56]. Additionally, some of their components may adversely affect the functional characteristics, taste, texture, and sensory appeal of subsequent formulations. Viable options for addressing this and incorporating WGs into diet would be completed through appropriate transformation into various other beneficial food forms, which would ensure the possibility of obtaining various value-added products. Although RGs are mostly used in fermented foods, the use of WGs as staple foods equally has a long history of human consumption [23]. Findings from epidemiological studies and discoveries, therefore, have triggered renewed interest among governmental bodies of different nations that WG should form part of cereal servings [24,57,58]. Table 1 summarizes common fermented WG products obtained through both solid-state fermentation (SSF) and liquid/submerged fermentation (SmF). While the former occurs in the absence or near-absence of free water, the latter occurs in the presence of free flowing water (more fluids compared to SSF). Subsequent fermented products are relatively few in contrast to numerous other studies reporting the use of RGs for similar food products, which necessitates further intensified research on the development of WG-fermented food products.

Due to the protective pericarp/seed coat, the fermentation process might be slightly hindered. Such has been reported in the literature and attributed to some of the antimicrobials and bioactive constituents in the seed coat that might mitigate the activity of fermenting microorganisms [55,90,93,94]. The protective pericarp layer of cereal tends to alter the diffusion of nutrients such as amino acids and sugars necessary for the growth of fermenting microorganisms. While this might result in a slightly higher pH and likely longer fermentation periods (in the absence of a starter culture), fermentation still modifies the phenolic constituents in WGs.

## 4. Impact of Fermentation on Phenolic Compounds in WGs

The fermentation process can have multiple effects on WG phenolics leading to modifications in inherent levels and/or formation of subsequent monomers or polymers. Adebo et al. [84] reported higher bioactive compounds (catechin, gallic acid, and quercetin) after fermentation in a study on ting from fermented WG-sorghum with a concurrent decrease in total flavonoid content (TFC), total tannin content (TNC), and total phenolic content (TPC). Reported decreases in levels of TPC, TFC, and TNC were attributed to degradation and hydrolysis of the phenolic compounds, while a corresponding increase in catechin, gallic acid, and quercetin was attributed to a release of these bioactive compounds after fermentation with *Lactobacillus* strains.

**Figure 3:**
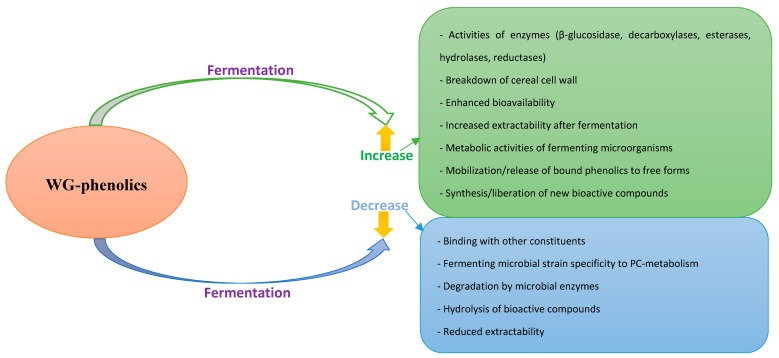
A summary of ways by which whole grain phenolic compounds are modified during fermentation.

Through fungal fermentation of WG-wheat into *tempe*, an increase in the sum of PAs was observed with up to a 382% increase in ferulic acid recorded after fermentation [92]. A similar trend of increase in investigated PCs and TPC during the fermentation of WG-*tempe* with *Rhizopus oryzae* RCK2012 had been reported earlier [91]. Salar et al. [62] equally reported an increase in TPC of the WG-millet-*koji* and attributed this to mobilization of PCs from their bound form to a free state through enzymes produced during fermentation. Similar authors earlier reported an increase in TPC during the fermentation of WG-maize [95], reportedly through the activities of β-glucosidase, which is capable of hydrolyzing phenolic phucosides to release free phenolics. Increased extractability of PCs, synthesis of new bioactive compounds, and consequent liberation of PCs due to structural breakdown of cereal cell walls have all been attributed to such increases in WG-PCs after fermentation (Table 2). Through metabolic activities of microbes, fermentation also induces structural breakdown of the cell wall, which leads to synthesis of various bioactive compounds [65]. Equally important are the roles of proteases, amylases, xylanases derived from fermenting microorganisms, and the cereal grain that contributes to modification of the grain and distorting of chemical bonds, which, consequently, releases bound phenolics (Figure 3).

During fermentation, PCs are metabolized and modified by fermenting organisms into other conjugates, glucosides, and/or related forms. Such a metabolism of PCs during fermentation have been reported to increase their bioavailability [104,105] and lead to generation of compounds that impact flavor [106,107]. Fermentation of sorghum into sourdough using LAB strains [singly and in two binary combinations (*L. plantarum* and *L. casei* or *L. fermentum* and *L. reuteri*)] was reported to have resulted in the metabolism of PAs, PA-esters, and flavonoid glucosides [108]. Most PCs in this study were metabolized and most notable were the transformation of caffeic acid → dihydrocaffeic acid, ethylcatechol, vinylcathechol, ferulic acid → dihydroferulic acid and naringenin-7-*O*-glucoside → naringenin, reportedly an indication of the presence of esterase (tannase), glucosidase, PA decarboxylase, and PA reductase [108]. The authors also suggested that the strains might have used different pathways for PA and flavonoid metabolism. Fermentation of WG-sorghum have also been reported to have led to the modification of PCs (catechin, gallic acid, and quercetin) into structurally related compounds, which were not identified [85]. The authors suggested that the observed modification could be attributed to decarboxylation, hydrolysis, and esterification reactions that might have occurred during fermentation [85]. In a study on the metabolism of PAs in whole wheat and rye malt sourdoughs, *L. plantarum* was observed to have metabolized free ferulic acid in wheat and rye malt sourdoughs, while a strain of *L. hammesii* (DSM 16381) metabolized syringic and vanillic acids and reduced levels of bound ferulic acid in wheat sourdoughs [102]. Co-fermentation of the LAB strains was also noted to have aided the conversion of resultant-free ferulic acid to dihydroferulic acid and volatile metabolites (vinyl-guaiacol and ethyl-guaiacol), which suggests that PA metabolism in sourdoughs is more enhanced by co-fermentation due to complementary metabolic activities [102]. Carboxylase, decarboxylase, esterase, and reductase activities in the LABs were reportedly responsible for PA metabolism in this study [102]. It should, however, be noted that such metabolism could lead to an increase in antimicrobial activities of resulting metabolic products [109], a decrease in antimicrobial activities [104,110], or no alteration in antimicrobial activity levels [108].

According to Gänzle [104], metabolism of PCs may involve the removal of noxious compounds as well as the release of hexosides as a source of metabolic energy. This metabolism could, however, be influenced by composition and intrinsic factors of the matrices/substrate and can, thus, influence the metabolic pathway, i.e., enzymatic activities can shift from decarboxylase action to reductase to glucosidase activity [111]. Glycosyl hydrolases have also been implicated as a group of enzymes responsible for such metabolism of PCs [104]. For example, *L. hammesii* was reported to have metabolized hydroxybenzoic acids in wheat but not in rye malt sourdoughs, which possibly reflects that the fermentation substrate influences the expression of enzymes active on PAs [111]. Likewise, in a study on sorghum sourdough, the accumulation of dihydrocaffeic acid by only *L. fermentum* indicates that decarboxylase and reductase enzymes of the other strains (*L. fermentum* and *L. plantarum*) have different substrate specificities [108]. The study of Gaur et al. [112] also suggests that availability of genes necessary for the metabolism of these PCs is also of importance and a significant contributor to the metabolic potential of fermenting microorganisms.

## 5. Impact of Fermentation on Antioxidant Activity in WGs

Antioxidants are endogenous or exogenous molecules that mitigate any form of oxidative/nitrosative stress or its consequences [113]. According to Slavin [114], the primary protective role of antioxidants in the body is through their reaction with free radicals. Antioxidants function as free radical scavengers, quenchers of singlet oxygen formation, and reducing agents [115,116] through their inhibitory activity of prooxidant enzymes. A potential mechanism by which PCs confer AA involves the induction of detoxification mechanisms through phase II conjugation reactions, which prevents the formation of carcinogens from precursors as well as by blocking the reaction of carcinogens with critical cellular macromolecules [117,118]. Phenolic compounds also modify some cellular signaling processes and donate an electron/transfer hydrogen atom to free radicals, activate endogenous antioxidant mechanisms, which increases the levels of antioxidant enzymes, and act as chelators of trace metals involved in free radical protection [116,119,120].

As evident in Table 3, most available studies in the literature investigating the influence of fermentation on phenolic compounds have majorly focused on AAs as its health benefit. This might be unsurprising as PCs, particularly PAs, have been reported as one of the most abundant metabolites of cereal crops with AAs [121,122,123]. While the role of other bioactive constituents in WGs cannot be disregarded, PCs equally play a huge role in the antioxidant properties it confers to WG-foods.

Although the majority of the studies reviewed herein reported increases in PCs, this is not always the case, as decreases in these health beneficial constituents have also been reported (Table 2). Studies on fermented WG-sorghum reported a decrease in TNC and TPC with this attributed to the ability of tannins to bind with proteins and other components, which reduces extractability as well as tannin degradation [79,85]. Investigations into the metabolism of sourdough by Ripari et al. [102] also suggested that reduction in some investigated PAs might be due to metabolism of PAs by lactic acid bacteria (LAB) and the activities of decarboxylases, esterases, and reductases. In the study of Dey and Kuhad [103] on fermentation of different WGs, both an increase and a decrease in TPC was observed. While increases alluded to enhanced bioavailability of cereal phenolics, a decrease observed in maize was associated with the specificity of the microbial strain to act on the PCs as well as the grain composition. The effect of the microbial activity on the levels of individual phenolics can differ, depending on the microbial strain. The genome of certain microorganisms might encode genes responsible for the metabolism and/or degradation of phenolic compounds while some do not [92,96,102]. This might, however, be difficult to ascertain or distinguish in spontaneous fermentation processes or back-slopping that is characterized by a wide range of fermenting microorganisms.

During the estimation of AA of food products, using more than one analytical method is better because food contains a myriad of constituents [92]. The frequently used techniques are spectrophotometric assays and the 2, 2′-Azino-bis (3-ethylbenzothiazoline-6-sulfate) (ABTS) (also called ABTS-radical cation depolarization) assay as well as the cupric-reducing antioxidant capacity (CUPRAC), 2,2-Diphenyl-1-picrylhydrazyl (DPPH) and ferric-reducing antioxidant power (FRAP) assay. Less frequently used techniques found in the course of this review are the lipid peroxidation technique adopting the thiobarbituric acid (TBA) assay, which was used to determine the TBA reactive substance from lipid peroxidation [101], as well as OH- and H_2_O_2_-scavenging assays. These are both concerning due to their role in causing tissue damage and cell death, and could combine with nucleotides to cause carcinogenesis [124].

Considering the general trend of increase in WG-PCs after fermentation and associated mechanisms, it could, thus, be hypothesized that this should be tantamount to an increase in AAs. While such increases were reported, some studies noted decreases in AAs of WG-fermented products. As documented by Ðordevic et al. [101] and Sun and Ho [125], possible explanations for this ambiguous relationship between AA and PCs are that: (i) quantified TPC values do not include other components that can equally confer AAs, (ii) synergy in a mixture makes AA not only dependent on antioxidant concentration but also on the structure and interactions among antioxidants, and (iii) different methods used for measuring AA based on different mechanisms may lead to different observations. Such an observation has also been buttressed by other authors suggesting that directly linking AAs in food and a responsible component might be somewhat difficult, as methods of extraction, identification, and/or quantification of AAs vary [126,127], which makes comparisons and, subsequently, extrapolating conclusions quite tricky.

General increases in AA of fermented foods have been attributed to a release of bound PC due to activities of hydrolytic enzymes and contents modulated during fermentation of a maize-based product and *koji* from millet [62,95]. A likely conversion of bound PCs into health-related components, a release of soluble phytochemicals and other non-PCs as well as increased extractability of AA-related PCs have equally been implicated to have led to an increase in AA during the fermentation of WGs into tempe, ting, and sourdough (from millet and rye) [65,69,85,92,97]. An addition to these could be that the fermentation process facilitated cleavage/dissociation of the bonds between PCs and other constituents leading to a release of PC-monomers, which yield AAs. Equally important and implicated in other studies are products of protein hydrolysis through proteolytic actions through fermentation, which could have led to components that contribute to increased PC and consequent antioxidant potential of fermented WGs. Available enzymes during fermentation and/or produced by fermenting microorganisms could also break down ester bonds, hydrolyse β-glucosidic bonds, and distort the hydroxyl groups in phenolic structures liberating free PCs and other antioxidant-related compounds. On the contrary, a decrease in AA after fermentation was attributed to modifications that influenced the extractability of compounds that confer AAs, especially the association between tannins, phenols, proteins, and other compounds in the grain [79].

Although in vitro studies reflect potential AAs of WG-fermented cereals, these in vitro techniques could underestimate physiological antioxidants, which necessitates in vivo studies. The use of in vivo models in investigating the influence of fermentation on AA is largely desirable. According to Benedetti et al. [128] and Alam et al. [129], in vivo protocols involve the administration of antioxidants to testing animals for a specified period of time, after which the animals are sacrificed, and blood or tissues are analyzed. Subsequently done are assays such as lipid peroxidation (LPO), thioredoxin reductase activities, and glutathione peroxidase (GSHPx) in human patients [128,130]. Although such in vivo studies are largely desirable, challenges related to ethical approvals, high costs, and daunting logistics have led to the adoption of in vitro techniques. Few studies are available on in vivo assays on fermented WG-cereal products with such studies focusing on AAs of the product. Breads made from WG-Kamut Khorasan wheat and WG-durum wheat were both reported to protect rat liver from oxidative stress [128]. An earlier study by similar authors reported a lower oxidative state in rats fed with experimental diets of sourdough bread for seven weeks [131].

Phenolic compounds usually occur in an esterified form linked to the cell wall matrix in the cereal bran and, as such, not readily available. Fermentation is considered a possible strategy to not only increase AAs but also to release the insoluble bound phenolic acids and, thus, to improve the poor bioavailability of grain phenolics [132]. This is particularly important as the antioxidant potential of WGs could be restricted by low availability of compounds during digestion. Not only does fermentation increases PCs and AA of WG-fermented products (Table 2 and Table 3), it also positively influences bioavailability, bio-accessibility, and PAs as demonstrated in a study on flours from WG-barley fermented with probiotic strains [96].

## 6. Future Perspective

Fermentation positively alters food quality, confers organoleptic characteristics, and improves phenolic constituents and antioxidant activity of WGs. Could this then translate to consumption of more whole grains? Possibly not, considering the grittiness and associated sensory challenges associated with whole grain foods. This might also contribute to fewer whole grain fermented foods as compared to those from refined grains. This is in tandem with a study on the consumption of WGs foods from brewers’ spent grain, which indicates that hereditary consumers of whole grain foods will be more receptive to its consumption as compared to their refined foods counterpart [133]. Some studies have also indicated barriers for consuming WG foods such as the lack of knowledge about its health benefits, challenges with cooking/preparation time, negative sensory perception, perceived cost, and the lack of availability of whole grains [134,135,136].

## 7. Conclusions

Increasing whole grain consumption should, therefore, be a target for health organizations with recommendations for intake proposed in many countries. As such, new strategies and partnerships between researchers, industry, and relevant agencies are further needed to promote whole grain consumption. Future studies are necessary in the area of phenolic compounds in fermented whole grains along with effective techniques such as whole genome sequencing to investigate genes responsible for the conversion of phenolic constituents and improvements in AAs. Such would largely assist in choosing starter cultures that would further improve the quality of fermented WG foods. Deeper investigation into the mechanisms of different forms of fermentation (solid state and liquid) on single/pure phenolic compounds (in isolation) and antioxidant activities should equally be explored. Additionally, studies are needed into the absorption and bioavailability of these phenolics in the gut, preferably through in vivo models.

## Figures and Tables

**Table 1:** Some reported fermented food products from whole grains.

Whole Grain(s)	Food	Type of Fermentation	Reference
Barley and oat	*Tempe*	SSF	Eklund-Jonsson et al. [59]
Maize	*Akamu*/*Ogi*	SSF	Oyarekua [60], Obinna-Echem et al. [61]
Millet	*Koji*	SSF	Salar et al. [62]
Millet	Probiotic drink	SmF	Di Stefano et al. [63]
Millet	Fermented milk	SmF	Sheela et al. [64]
Millet	Sourdough bread	SSF	Wang et al. [65]
Oat	Fermented oat	SSF	Wu et al. [66]
Oat, wheat	Bread	SSF	Gamel et al. [67]
Quinoa	Yoghurt	SmF	Zannini et al. [68]
Quinoa, wheat	Fermented product	SSF	Ayyash et al. [69]
Rye, oat, wheat	Bread	SSF	Buddrick et al. [70]
Rye, wheat	Sourdough bread	SSF	Koistinen et al. [71]
Rye	Bread	SSF	Johansson et al. [72], Raninen et al. [73]
Rye	Porridge	SSF	Lee et al. [74]
Rye	Sourdough bread	SSF	Beckmann et al. [75], Zamaratskaia et al. [76]
Sorghum	*Burukutu*	SmF	Ikediobi et al. [77]
Sorghum	Fermented balls	SSF	Ragaee and Abdel-Aal [78]
Sorghum	Fermented porridge	SSF	Dlamini et al. [79]
Sorghum	*Injera*	SSF	Taylor and Taylor [80]
Sorghum	*Ogi*	SmF	Akingbala et al. [81]
Sorghum	*Omuramba*	SmF	Mukuru et al. [82]
Sorghum	*Ting*	SSF	Kruger et al. [83], Adebo et al. [84,85]
Sorghum	*Uji*	SmF	Taylor and Taylor [80]
Tef	*Injera*	SSF	Tamene et al. [86]
Wheat	*Boza*	SmF	Gotcheva et al. [87]
Wheat	Bread	SSF	Mustafa and Adem [88], Struyf et al. [89]
Wheat	Sourdough bread	SSF	García-Mantrana et al. [90]
Wheat	*Tempe*	SSF	Dey and Kuhad [91], Starzyńska-Janiszewska et al. [92]

SSF—solid-state fermentation. SmF—submerged/liquid fermentation.

**Table 2:** Documented studies on the effect of fermentation on phenolics of whole grains.

Whole Grain	Fermented Product	Phenolics Investigated	Analytical Method	Findings	References
Barley and oat groat	Fermented product	Free and bound PAs	Colorimetric; HPLC	Increase in total content of bound PAs in flours from WG-barley related to increased content of bound *p*-coumaric acid, ferulic acid, and dimers of ferulic acid (5,5′- diferulic, 8-*o*-4-diferulic, and 8,5′-diferulic acids).	Hole et al. [96]
Maize	Fermented product	TPC	Colorimetric	Increase in TPC after fermentation attributed to the activities of β-glucosidase, capable of hydrolyzing phenolic phucosides to release free phenolics	Salar et al. [95]
Millet	*Koji*	TPC	Colorimetric	Increase in TPC of fermented product due to mobilization of PCs from their bound form to a free state through enzymes produced during fermentation	Salar et al. [62]
Millet	Sourdough bread	TPC	Colorimetric	Increase and decrease in soluble and bound phenolic content. Slight decrease in TPC observed. Increment of soluble phenolic content may be due to acidification, production of hydrolytic enzymes by LAB, and/or activation of indigenous cereal enzymes, which broke down the bran cell wall structure	Wang et al. [65]
Quinoa, wheat	Fermented product	TPC	Colorimetric	Increase in TPC may be attributed to hydrolytic activities (e.g., esterases) of Bifidobacteria strains that released more PCs via the hydrolysis of complexed forms, possibly the synthesis of new bioactive compounds detected as PCs	Ayyash et al. [69]
Rye	Baked sourdough	TPC, PAs	Colorimetric, HPLC	Fermentation phase more than doubled the levels of easily extractable PCs	Liukkonen et al. [97]
Rye	Sourdough	TPC, PAs	Colorimetric, HPLC	Increased level of total PCs due to increases in methanol-extractable PCs. Modification in levels of bioactive compounds during fermentation by the metabolic activity of microbes. Fermentation-induced structural breakdown of cereal cell walls might have also occurred and led to liberation and/or synthesis of various bioactive compounds	Katina et al. [98]
Rye, wheat	Whole meal bread	PAs	HPLC	Increase in PAs due to activities of phenolic acid esterases during the fermentation stage	Skrajda-Brdak et al. [99]
Sorghum	Fermented porridge	TPC, TNC	Colorimetric	Reduction in TNC and TPC. Reduction in TNC could be due to binding of tannins with protein and other components, which reduces their extractability and tannin degradation by microbial enzymes	Dlamini et al. [79]
Sorghum	Fermented product	TPC, TNC	Colorimetric	Increase in TPC, decrease in TNC	Mohapatra et al. [100]
Sorghum	*Ting*	Flavonoids, PA, TFC, TNC, TPC	Colorimetric, LC-MS/MS	Decrease in TFC, TNC, and TPC attributed to possible degradation of PCs and hydrolysis of bioactive compounds. Breakdown of tannin-related compounds to lower molecular weight compounds, which affected extractability. Increase in PA and flavonoids could be due to decarboxylation, hydrolysis, microbial oxidation, and reduction as well as esterification reactions that occurred during fermentation	Adebo et al. [84,85]
Wheat	Fermented product	TPC	Colorimetric	Increase in TPC through modification in levels of bioactive compounds during fermentation by the metabolic activity of microbes	Ðordevic et al. [101]
Wheat	Sourdough	PAs	LC-MS/MS, UPLC	Degradation, reduction of some PAs and content of some remain unchanged. Release of PAs from bound fraction, metabolism of PA by LAB strains and action of enzymes (decarboxylases, esterases, and reductases)	Ripari et al. [102]
Wheat	*Tempe*	TPC, PCs	Colorimetric, TLC, UPLC	Increase in TPC after fermentation, possibly due to release of bound compounds from the wheat matrix	Dey and Kuhad [91]
Wheat	*Tempe*	Free and condensed PAs	HPLC	Increase in the sum of PA could be linked to an increase in their extractability after fermentation	Starzyńska-Janiszewska et al. [92]
Wheat, brown rice, maize, oat	Fermented product	TPC, PAs	Colorimetric, HPLC	TPC of all fermented samples increased except for *Rhizopus oligosporus* fermented maize. Increase as well as decrease in PA levels. Decreases was attributed to strain/specie specificity and/or grain composition. General increases were alluded to enhanced bioavailability of cereal phenolics.	Dey and Kuhad [103]

HPLC—high performance liquid chromatography. LAB—lactic acid bacteria. LC-MS/MS – liquid chromatography tandem mass spectrometry. PA—phenolic acid. PC—phenolic compound. TFC—total flavonoid content. TLC—thin layer chromatography. TNC—total tannin content. TPC—total phenolic content. UPLC—ultra high-performance liquid chromatography.

**Table 3:** Documented studies on the effect of fermentation on antioxidant activity of whole grains.

Whole Grain	Fermented Product	Assay	Mechanism(s) Reported	References
Maize	Fermented product	ABTS, DPPH	Increase in ABTS and DPPH due to the role of the hydrolytic enzyme that released/mobilized bound polyphenolic compounds, which enhanced AAs.	Salar et al. [95]
Millet	*Koji*	ABTS, DPPH	*Koji* showed increased scavenging of ABTS and DPPH radicals due to the release of a bound form of phytochemicals present and high levels of TPC modulated during fermentation.	Salar et al. [62]
Millet	Sourdough bread	DPPH	Increase in DPPH radical inhibition after sourdough fermentation. The conversion of bound to soluble PCs improved the health-related functionality of the final products.	Wang et al. [65]
Quinoa, wheat	Fermented product	ABTS, DPPH	An increase in ABTS and DPPH values was attributed to the soluble phytochemicals released during fermentation and to bioactive peptides formed as a result of proteolytic activity.	Ayyash et al. [69]
Rye	Baked sourdough	DPPH	The fermentation stage increased AA likely due to an increased level of extractable PCs.	Liukkonen et al. [97]
Sorghum	Fermented porridge	ABTS, DPPH	Reduction in antioxidant levels after fermentation attributed to changes during processing that affected the extraction of total phenols and tannins. Such changes were hypothesized to have likely involved associations between the tannins, phenols, proteins, and other compounds in the grain.	Dlamini et al. [79]
Sorghum	Fermented product	CUPRAC, DPPH	Increase in AAs investigated.	Mohapatra et al. [100]
Sorghum	*Ting*	ABTS	Increase in AA due to regenerated and released bioactive compounds (including non-phenolic components after fermentation with the *L. fermentum* strains), which might have contributed to the radical scavenging properties of the product.	Adebo et al. [85]
Wheat	Fermented product	DPPH, FRAP, TBA	Increase in the investigated AAs.	Ðordevic et al. [101]
Wheat	*Tempe*	ABTS, DPPH, FRAP, HP-scavenging and OH-scavenging assays	Increase in antioxidant properties investigated attributed to the composition of PCs, unidentified compounds, and other water-soluble bioactive compounds like small peptides and xylo-oligosaccharides produced during fermentation.	Dey and Kuhad [91]
Wheat	*Tempe*	ABTS, OH-scavenging and FCRS-RP assays	Increase in soluble antioxidant potential as fermentation increased extractable antiradical activity scavenging potential, which might be due to the release of peptides and other compounds during fermentation.	Starzyńska-Janiszewska et al. [92]
Wheat, brown rice, maize, oat	Fermented product	ABTS, DPPH	Both ABTS and DPPH scavenging properties were enhanced after fermentation of the WG-cereals by all the four micro-organisms (except *R. oligosporus*-fermented maize). Increases related to release of more soluble bioactive compounds, such as peptides and oligosaccharides.	Dey and Kuhad [103]

ABTS-2,2′-azino-bis(3-ethylbenzothiazoline-6-sulfonic acid). CUPRAC—cupric reducing antioxidant capacity. DPPH—2,2-diphenyl-1-picrylhydrazyl. FCRS-RP—Folin-Ciocalteu reacting substances-reducing power. FRAP—ferric reducing antioxidant property. HP—hydrogen peroxide. HPLC—high performance liquid chromatography. OH—hydroxyl.
